# The PED-t trial protocol: The effect of physical exercise –and dietary therapy compared with cognitive behavior therapy in treatment of bulimia nervosa and binge eating disorder

**DOI:** 10.1186/s12888-017-1312-4

**Published:** 2017-05-12

**Authors:** Therese Fostervold Mathisen, Jan H. Rosenvinge, Gunn Pettersen, Oddgeir Friborg, KariAnne Vrabel, Solfrid Bratland-Sanda, Mette Svendsen, Trine Stensrud, Maria Bakland, Rolf Wynn, Jorunn Sundgot-Borgen

**Affiliations:** 1 Department of Sports Medicine, Norwegian School of Sport Sciences, Sognsvegen 220, 0806 Oslo, Norway; 2 Department of Psychology, UiT -The Arctic University of Norway, Faculty of Health Sciences, 9037 Tromsø, Norway; 3 Department of Health and Caring Sciences, UiT -The Arctic University of Norway, Faculty of Health Sciences, 9073 Tromsø, Norway; 4 Research Institute of Modum Bad, Badeveien 287, 3370 Vikersund, Norway; 5 University College of Southeast Norway, Bø Postboks 235, 3603 Kongsberg, Norway; 6 Department of Preventive Medicine, Oslo University Hospital, Kirkeveien 166, 0407 Oslo, Norway; 7 Department of Clinical Medicine, UiT -The Arctic University of Norway, Faculty of Health Sciences, 9037 Tromsø, Norway

**Keywords:** Eating disorders, RCT, Physical exercise, Dietary therapy, CBT, Treatment outcome, Physical fitness, Bone mineral density, Resistance exercise

## Abstract

**Background:**

Sufferers from bulimia nervosa (BN) and binge eating disorder (BED) underestimate the severity risk of their illness and, therefore, postpone seeking professional help for years. Moreover, less than one in five actually seek professional help and only 50% respond to current treatments, such as cognitive behavioral therapy (CBT). The impetus for the present trial is to explore a novel combination treatment approach adapted from physical exercise- and dietary therapy (PED-t). The therapeutic underpinnings of these separate treatment components are well-known, but their combination to treat BN and BED have never been previously tested. The purpose of this paper is to provide the rationale for this new treatment approach and to outline the specific methods and procedures.

**Methods:**

The PED-t trial uses a prospective randomized controlled design. It allocates women between 18 and 40 years (BMI range 17.5–35.0) to groups consisting of 5–8 members who receive either CBT or PED-t for 16 weeks. Excess participants are allocated to a waiting list control group condition. All participants are assessed at baseline, post-treatment, 6, 12 and 24 months’ post-follow-up, respectively, and monitored for changes in biological, psychological and therapy process variables. The primary outcome relates to the ED symptom severity, while secondary outcomes relates to treatment effects on physical health, treatment satisfaction, therapeutic alliance, and cost-effectiveness. We aim to disseminate the results in high-impact journals, preferable open access, and at international conferences.

**Discussion:**

We expect that the new treatment will perform equal to CBT in terms of behavioral and psychological symptoms, but better in terms of reducing somatic symptoms and complications. We also expect that the new treatment will improve physical fitness and thereby, quality of life. Hence, the new treatment will add to the portfolio of evidence-based therapies and thereby provide a good treatment alternative for females with BN and BED.

**Trial registration:**

Prospectively registered in REC the 16th of December 2013 with the identifier number 2013/1871, and in Clinical Trials the 17th of February 2014 with the identifier number NCT02079935.

## Background

The present paper reports on an ongoing, new treatment for bulimia nervosa (BN) and binge eating disorders (BED). Binge eating disrupts normal eating patterns and introduces vicious circles of fasting and binging that causes a chaotic energy intake. Also, binge eating causes an energy surplus, which those with BN compensate for by purging, *eg.* vomiting, using laxatives, diuretics, or enemas, misusing medications or exercising in an extreme or rigid manner, all which can change body metabolism. It is noteworthy that such changes may also be the result of physical inactivity, which is frequently observed particularly among sufferers from BED.

Binge eating and compensatory behaviors can raise the medical severity of BN and BED by means of hypokalemia, that may elicit inter-current infections as well as cardiac complications, diseases or arrest, or it may affect glucose, insulin and lipid levels that increases the risk of type-2 diabetes [1–5]. A combination of rigid exercising and eating may over time inflict chronic low energy availability and raise the risk for low bone mineral density, and ultimately osteoporosis [6, 7]. The non-medical severity comprises personal and familial burdens. A slightly raised standardized mortality rate is due to both medical and non-medical burdens [8–10]. According to a recent systematic review, the societal health care costs range from €888–18,283 for BN and €1762–2902 for BED [8–10].

Most sufferers do not acknowledge the clinical severity and the medical and non-medical risks of eating disorders (ED), as 80–94% of people with BN and BED never seek professional help or delay it 4–5 years [11–15]. Effective treatment of BN and BED is considerably hampered by this incongruence, which may be circumvented by offering treatment options outside the contexts of traditional health services or improving access to such options. Among those who do enter treatment, a protracted course of illness is typically seen among every third patient [16]. Important reasons are early drop-out due to a failure to engage patients into treatment and lack of rapid symptom changes or symptom coping, thereby lowering patients’ motivation and self-efficacy [17].

In most treatment guidelines, cognitive-behavioral therapy (CBT) is regarded as the treatment-of-choice for BN [18]. Clinical targets in CBT include weakening the strength of core beliefs about low self-worth and compensatory beliefs about the need to control food intake, body weight and shape. Use of stimulus-control procedures to reduce the frequency of disordered eating, *ie.* bingeing and/or purging [19] is also central. Studies, systematic reviews and meta-analyses show promising effects of CBT for BED, and intermediate effects for BN, but the methodological quality of studies is low to moderate [20–22] even when often observed comorbid conditions are incorporated in the treatment [23–25]. New treatment approaches are called for because up to 50% of patients do not respond to CBT, even when the therapy is especially designed for ED [26].

Guided physical exercise (PE) may facilitate regulation of negative emotions [27], yet guided PE is rarely used in clinics [28] due to a fear of reinforcing the excessive exercise used to compensate for bingeing. A previous randomized controlled trial has shown that guided PE is as effective as CBT in alleviating BN symptoms [29]. The present study aims to replicate and expand on these findings.

Dietary therapy (DT) and counseling alone to correct the chaotic eating pattern has unclear empirical support [5, 30], but one review indicate some support to a possible *additive* effects of DT with CBT [31]. The present trial is the first one to examine the additive effects of DT and PE (PED-t) compared to CBT alone, and using a group therapy format.

Therapeutic alliance is an important moderator (or even mediator) of treatment effects in general; however, for EDs its impact is less understood [18, 32, 33]. High treatment expectations and installment of hope seems to predict treatment alliance in anorexia nervosa, but the relationship is uncertain for BN and BED [34]. Recent literature reviews have shown inconsistent findings across ED diagnoses, treatments, patient age groups and time (*eg*. early, mid or late) of assessment [31, 35, 36]. We hence included measures of alliance and group climate after every CBT and PED-t session to study the temporal order between these factors and response in both treatment arms [31].

### Hypotheses and predictions

This trial will test six hypotheses:

1) Comparison of treatment arms: a) PED-t and CBT have comparable effects in terms of less symptoms of eating pathology, b) both perform better than a wait-list control group in reducing binge eating and/or purging, and c) both improve dietary intake.

2) Compared with CBT, the PED-t intervention produces a more rapid treatment response.

3) By improving physical strength and endurance, body composition, bone mass, and the nutritional and hormonal status, PED-t will surpass CBT in reducing the number and severity of medical complications.

4) Positive early changes in therapeutic alliance scores and group cohesiveness partly mediate effects of PED-t and CBT.

5) PED-t surpasses CBT in terms of a lower dropout rate and higher patient satisfaction with treatment.

6) The direct treatment costs are comparable for PED-t and CBT, but the indirect costs of PED-t are expected to be lower.

We predict that the PED-t combination will serve as an effective treatment method for BN and BED because the preoccupations of exercise and diets are transformed away from being ED-symptoms and toward functional coping and self-regulative activities. We also predict that the treatment effect of PED-t will be rapid and strong, defined according to a systematic review and meta-analysis [37], and the best empirically derived predictors of sustained remission at 6 and 12 months follow-up [38, 39] *ie*. as a 25% reduction in depression scores and ≤3 binge eating episodes a week during the first four weeks of treatment. Given that our novel treatment (PED-t) targets clinical features that occupy the sufferers’ minds, we expect a high level of treatment engagement, motivation and compliance. Such accomplishments may facilitate a stronger therapeutic alliance [40].

## Methods and design

**Fig. 1:**
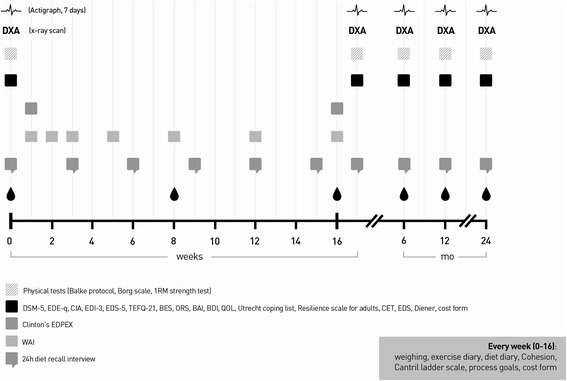
Overview of measures and measurement points. Abbreviations are explained by full name in Table 4

This randomized controlled trial includes three groups: participants are randomized to either the CBT or the PED-t treatment group, while participants having to wait represent the control group condition. After 16–20 weeks, these are randomized to either treatment arm. The treatments are delivered to groups consisting of 5–8 participants. It includes 20 therapy sessions stretching across 16 weeks. All outcome variables are measured five times: pre, post, 6, 12 and 24 months (Fig. 1). Mediator/moderator data (*ie*. therapy process variables) are collected after each treatment session, which yields excellent statistical power for conducting growth curve modeling, nuanced mediation or moderation analyses.

Treatment groups run through 2014–2016. The last follow up assessment is scheduled in December 2018. A detailed treatment manual for the new treatment method (PED-t) can be provided upon request. All treatment sessions are arranged at the Norwegian School of Sport Sciences in Oslo, Norway (NSSS).

### Inclusion and exclusion criteria

Included are women aged 18–40 years with a BMI in the range of 17.5–35, a DSM-5 diagnosis of BN or BED with duration of at least 3 months, and with mild to severe symptoms (minimum one episode per week of compensatory behaviors or binge eating, respectively) [41]. A signed letter from the women’s general practitioner (GP) confirming their suitability for the study is required for final enrollment.

Women not eligible are those who are or plan to become pregnant during the study period and those who are competitive athletes. Also excluded are those with a concurrent severe axis I and/or axis II mental disorder obviously in need of other treatments options not focusing on the ED. To prevent effect diffusions we also excluded individuals who have received CBT for ED during the last two years before the trial.

### Recruitment

Study- and contact information is distributed through GP’s, magazines and websites of the ED patient organizations, newspaper ads, national TV, social media, and posters.

**Fig. 2:**
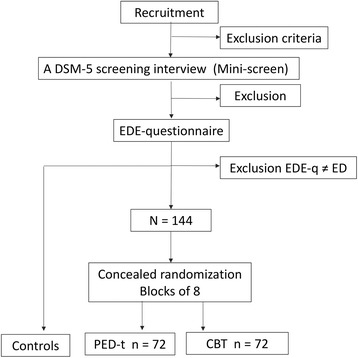
Recruitment, screening and randomization of participants

Individuals calling by phone are informed about the project purpose, and for those who pass the inclusion criteria, a diagnostic screening is conducted using the Mini-International Neuropsychiatric Interview screening [42] and the Eating Disorder Examination (EDE-q) [41, 43, 44] (Fig. 2). Final inclusion is based on three written and signed declarations, *ie,* the informed consent by the woman to take part in the trial and the assessment procedures herein, a declaration of mutual secrecy about personal information revealed in the group treatment sessions, and a signed consent from their GP that they are medically fit to participate. These documents are returned personally upon the first visit to the NSSS.

### Randomization

The participants complete the baseline measures before randomization. We use block randomization of size eight (https://www.randomizer.org/) to ensure equal sized treatment arms. A project-independent fellow worker allocated eligible participants to either treatment arm according to a concealed randomization list. Researchers and test personnel in the study are blind with regard to group allocation, but due to the nature of the two treatment procedures, therapists and participants are obviously not blinded to the treatment allocation. The participants are informed about their group allocation after completing the baseline measures.

### Safety procedures

A stop-procedure is activated if included subjects display a worsening of ED symptoms (eg. a BMI < 17.0 and/or rapid and significant weight loss of more than 3 kg from normal weight), severe depression or if severe osteoporosis is identified among those randomized to PED-t. Individuals excluded for any of these reasons are medically evaluated for admittance to health care services. Further: if participants report not to have been eating properly before exercise sessions in the PED-t groups, or if feeling ill, we tell the participant not to participate in the physical test or the exercise session that day. During the pre- and post-tests, if participants reports severe purging or restrictive eating in the days before, no physical tests will be performed. A defibrillator is available in the lab where physical tests are held, and a medical health care team is available next house if help is needed.

### Statistical power and analyses

Analysis of covariance (ANCOVA) is used to raise statistical power. The effect size of the covariates is set to *R* ^*2*^ = 25%, alpha = .05 and 1-beta = .80. The CBT treatment effect for EDs using the Eating Disorder Examination Questionnaire is about *d* = 1.30 [45]. A small change (*d* = 0.30) in the wait-list condition requires 14 subjects in each group (total *N* = 28), but since the difference between the PED-t and CBT treatment conditions is of primary interest a non-inferiority margin of *d* = 0.45 is considered as clinically relevant [46]. This requires a sample size of 62 + 62 subjects, increasing to 72 + 72 when adjusting for the group therapy factor (intra-class correlation = .05, design factor 1.16). The longitudinal data will be analyzed using multilevel regression models to accommodate for dependency in the repeated data within subjects and within groups. The maximum likelihood procedure uses all information available, thus, handling dropout well. Multiple imputation will be used to impute missing values. Follow-up data is analyzed with mixed model regression to estimate correct between-group treatment effects adjusted for within-group variance components related to patients and groups. Moderator variables will be included to analyze whether any treatment effects are modified by variables as motivation, therapist alliance or group cohesiveness. If treatment arms do not differ in outcome, combined latent class growth curve modeling will be conducted to analyze reasons for individual differences at startup and in the rate of change across time.

### Interventions

#### The physical exercise and the dietary therapy (PED-t)

##### Physical exercise

The PED-t is a treatment method particularly designed for BN and BED by our research group, and adheres to recent guidelines developed from systematic reviews to successfully use therapist guided physical exercise [47], and recommendations for a minimal training volume to accomplish a health benefit [48–51].

The intervention aims to (re)establish healthy eating and exercise routines, to change body ideals by focusing on the body’s functionality rather than body appearance and to provide knowledge about the harmful metabolic effects of swings between dieting and food craving. Education about harmful effects of unhealthy behavior is given and how basic and sports related nutritional needs may be balanced.

Three physical trainers and three dieticians conduct the intervention, all holding a master degree in sport sciences and having about three years of practice with supervised exercise. To qualify as a PED-t therapist the physical trainers hold a bachelor’s degree in physical exercise and health or exercise medicine.

The weekly training program in PED-t aims to establish a healthy volume of physical activities, emphasizing the training principles of progression and variation. The exercise program consists of three weekly exercise sessions, each of 40–60 min’ duration. Two sessions are resistance exercise of which one is supervised. The third exercise session consists of unsupervised pyramid interval running, involving shifts between intensive work-periods and active rest-periods with progressive duration (from one minute work period to 4 min of work period).

There are three reasons to focus on giving the participants experience with resistance exercise. First, regular resistance exercise may improve metabolism and bone mineral density, and increase muscular mass and strength [49, 52–54]. Change in body composition further increases the potential for improvement in energy metabolism, weight control and metabolic status [52, 55]. Second, the repeated bouts of supervised resistance exercise, involving technical guiding/corrections while lifting may improve body posture and awareness. Third, strength training involves easily coordinated movements, which may increase the likelihood of positive experience of mastery and weekly progression in performance. These bodily experiences during and after a period with exercise training, have potential to improve body image, self-perception, affect regulation and feeling of coping [56–60]. Intensive work periods, followed by active recovery periods, as practiced with interval training may improve cardiovascular health more effectively and with a lower risk compared to the extended exercise regimen often seen in ED [49, 61–64].

The physical strength-training program intends to improve maximal strength and growth. It is divided into five microcycles with both linear and daily variation in training load [65]. Linear progression involves increased intensity and reduction of training volume over a period of time, with the objective to increase maximal strength. Each week consists of one supervised heavy load, and one unsupervised medium load session. In the former, the number of repetition maximum (RM) increases progressively during the 16 weeks, while in the medium load session, relative load is kept constant at 10RM.

The exercises in the resistance exercise program are squats in smith-machine, lunges with dumbbells, seated dumbbell shoulder press, bench press, latissimus pulldown and seated row in cable machine. The intensity and work periods in the interval running program, follows a traditional pyramid structure performed with progressive interval periods and active rest periods, and then repeated in reverse. After a 10 min warm up, the interval periods are initiated by the first work period of 1 min and a 30 s active rest period (lower intensity), followed by the next work periods of 2 and 3 min and their corresponding rest periods of 60 and 90 s respectively. During the last six weeks, an extra interval period adds to the program with 4 min of work and 2 min of active rest (Table 1).

**Table 1:** Overview of the exercise module of the PED-t treatment arm

		SUPERVISED EXERCISE	UNSUPERVISED EXERCISE	
Week	Microcycle	Resistance exercise	Interval running	Resistance exercise
1–3	1	10 RM	Pyramid interval	10 RM
4–7	2	8 RM	Pyramid interval	10 RM
8–11	3	6 RM	Pyramid interval	10 RM
12–14	4	4 RM	Pyramid interval	10 RM
15–16	5	2 RM	Pyramid interval	10 RM

Resistance load is given as number of repetition maximum (RM)

During the mid-period (week 8–11) a supervised fourth, weekly group exercise session is introduced, leading the participants to do both the resistance exercise sessions unsupervised. The intention of introducing the participants to the fourth, additional exercise sessions is to inspire them to find a variety of training modalities that can improve physical fitness and promote training joy. Further, the intention with the group sessions is also to make them more experienced in exercising together with others, supervised by an instructor. The four sessions are Total body Resistance eXercise (TRX), suspension training or Cat slide exercise, one boot camp session, one indoor cycling class, and one combat inspired session.

##### Dietary therapy

The dietary therapy consists of three modules (Table 2), aiming to re-establish healthy dietary routines through weekly lectures, and on discussions related to the weekly topics and the experiences by the participants. Between every session, the participants register the meals they are consuming (pen and paper), and work on individual tasks related to dietary routines (increase number of meals per day, increase volume of food in each meal, improve the composition of the meal etc.).

**Table 2:** Overview of the content of the dietary module of the PED-t treatment arm

Module	Therapy session	Targets	Main content
1	1–5	Dietary routines & structure	Meal frequency
			Portion size
			Eating situation
			Exercise theory
			Repetition and summary
2	6–17	Nutritional knowledge & practical skills	Energy needs
			Daily routines
			Nutrients
			Nutritional labels
			Impulsive food shopping
			Exercise theory
			Sports nutrition
			Repetition and summary
3	18–20	Summary of future plans	Reflections, repetition and summary
			Presenting a personal plan for the future (exercise, diet, daily routines)

#### The cognitive behavioral therapy (CBT)

Our manual-based CBT has a group format, and rests on the transdiagnostic model of generic core ED-pathology across ED-diagnoses [45]. The treatment runs through four stages (Table 3). All sessions are videotaped and coded according to a CBT manual adherence form [66]. Psychologists who are experienced with CBT and EDs run the CBT treatment.

**Table 3:** Overview of the cognitive behavior therapy (CBT) module

Stages	Therapy session	Targets	Main content
1	1–4	Engagement, preparation and early behavior change	Educate about the nature of CBT and how the therapist and the participants work together
			Engage the participants in the treatment.
			Develop a case formulation for each participant.
			Strategies to take control over the behavioral symptoms of BN and BED
2	5–6	Monitoring and evaluating progress and barriers to change	A detailed review of progress so far, and to identify barriers to change
3	7–16	Modifying the core pathology of ED	Reduce the over-evaluation of weight and shape
			Address extreme dieting, binge eating, and purging
4	17–20	Consolidating change and relapse prevention	Secure that progress is maintained after treatment end

#### Measures and variables

Standardized instruments with good psychometric quality and high clinical utility are included throughout. Count variables measure physical injury and illness [67], and the number of participants meeting DSM-5 criteria for BN or BED [41], respectively. Psychological measures comprise both positive and negative clinical features (Table 4). Since variables of the same measurement domain (*eg*., eating pathology) will likely be highly multicolinear, these variables will not be combined in the same regression analysis.

**Table 4:** Overview of the psychological measures in the PED-t trial

Eating Disorder Examination-Questionnaire (EDE-q) [95]	Binge Eating Scale (BES) [96]
Clinical Impairment Assessment (CIA) [95]	Beck Anxiety Inventory (BAI) [97]
Eating Disorder Inventory-3 (EDI-3) [98]	Three-Factor Eating Questionnaire (TFEQ-21) [99]
Eating Disturbance Scale (EDS) [100]	Beck Depression Inventory (BDI) [101]
Subjective well-being scale [102]	Cantril’s Ladder Scale [103]
Utrecht Coping List [104]	Resilience Scale for Adults [105]
Outcome Rating Scale (ORS) [106]	Oslo Sports Trauma Research Center questionnaire on health problems (OSTRC) [67]

Biological measures acknowledged as gold standard methods for assessing physical fitness as well as body composition and bone health [68–70] are used. They comprise changes in blood pressure, serum ferritin, total, high, and low cholesterol, respectively, triglycerides, Apo A, Apo B, vitamin D, folic acid, leptin, insulin CTX-1 and PTH, estradiol, progesterone, TSH, T3, T4, FSH, LH, and cortisol. Also included are objective measures of physical activity (ActigrapgGT3X) [71], a cardio pulmonary exercise test (CPET) to screen for aerobic physical fitness (an incremental modified Balke protocol and Borg Scale) [72–74], muscular strength (1RM test on three selected events -squats, bench press, seated row) [69], resting metabolic rate, bone mineral density (BMD), body fat and lean body weight. Body composition is measured using **d**ual-**e**nergy **x**-ray **a**bsorptiometry (DXA) (Lunar iDXA, GE Healthcare, enCORE Software, Version 14.10.022) performing a three site scan (lumbar L2-L4, femoral neck, −trochanter and –shaft, and whole body scan) and analysis procedure according to the guidelines for best practice [75]. Physical tests and DXA-measurements are conducted in the lab at the NSSS, and blood samples are collected by qualified lab personnel and stored frozen until analyzed in a certified lab.

Reasons for physical exercise is measured using the Exercise Dependence Scale [76], and the Compulsive Exercise Test [77]. Throughout the 20 weeks of the physical and dietary therapy, program a training diary records intensity, type of training, and time. Dietary intake and energy/nutrient status is measured through a 24-h diet-recall interview (pre, week 3, 6, 9, 12, 16 and post), weight change (pre/post and separate weekly weigh-ins). Blood samples are taken in week 0, 8, and 17, respectively.

Patient satisfaction is measured using the “Expectations and experiences of ED-treatment” scale [78]. Qualitative approaches, such as in-depth interviews, may give additional insights into ED-patients’ perspectives and satisfaction with treatment [79, 80]. Therefore, a sufficient number of participants to meet data saturation criteria [81] are qualitatively interviewed about a) the immediate overall satisfaction with particular therapy sessions, the therapy process and outcomes, b) the recovery process; and c) the extent to which participants experience long-term treatment benefits. Interview data are analyzed in four steps within the framework of systematic text condensation [81]. Direct and indirect costs associated with treatment and follow-up are recorded prospectively.

Mediator variables comprise the “Working Alliance Inventory” [82] and the “Coerciveness” subscale from the “Therapeutic Factor Inventory” scale [83], both showing good psychometric qualities [84, 85].

Participants not continuing in the study will be compared to those who do continue by examining for differences in the pretest outcome measures.

## Discussion

Support to our hypotheses and predictions will provide a platform for enlarging the portfolio of evidence-supported treatments for BN and BED. Enlarging this portfolio is a major achievement given the complex nature of these disorders. An important additional task is to examine which patient, treatment or common therapeutic factors that facilitate a stronger response to cognitive behavioral treatment versus dietary/physical therapy. By bringing ahead knowledge of who responds best to which kind of treatment, we may offer help to a larger proportion of sufferers of ED.

The present trial also offers a possibility of exploring generic therapeutic mechanisms, *eg.* therapeutic alliance, group cohesion or experience of universality among group members facilitating treatment effects. For EDs the impact of such mechanisms in CBT treatment is less understood [18, 31–33, 35, 36] in particular with respect to BN and BED [34], and for obvious reasons completely unknown with respect to the novel physical exercise and dietary treatment program. The exploring of the impact of such mechanisms imply a search for the mediation effects and when such effects occur during the course of treatment. This knowledge may pave the way for future research into the effects of treatment modules, the succession of modules and the possibility of shortening the treatment length without cutting down on treatment intensity.

In principle, as effective treatments can be unaffordable and intolerable, it is essential to capture the patient satisfaction aspects. The limited number of studies of patients’ satisfaction with treatment have used biased retrospective recalls of treatment modalities rather than methods [11, 86, 87]. In contrast, we use prospective quantitative and qualitative measures to study how satisfaction might be associated with pretreatment expectations, treatment elements, or generic factors in the two treatment arms.

The level of actual knowledge about the costs and benefits of treatments does not meet health authorities’ need to control the health budgets. A recent review located only one CBT study on EDs, also flawed by retrospective recall of direct costs only [8]. The present trial adds to the basis of knowledge by prospective recording of direct and indirect illness-related costs.

In recent years, dissemination has been highly at focus with respect to CBT [88], showing that CBT can be delivered by health professionals other than heavily trained and payed psychologists and psychiatrists. An affordable and tolerable new program with an equal or better effect than CBT would represent major societal benefits in the sense that the PED-t can be delivered by new groups of professionals, *eg*. exercise therapists and registered dietitians, hence a possibility of reaching out to new segments of those sufferers who are reluctant to seek help through the health care services.

However, a concern can be raised about treatment effectiveness, *ie*. how well the PED-t might perform in clinical settings. Two deviations from such settings should be mentioned, i.e. the failure to offer booster sessions to consolidate changes during treatment, and to exclude severe comorbid conditions frequently found among patients with BN and BED [89–93], notably anxiety, depression, personality disorders, and active substance abuse. Regrettably, on the other hand, a long waiting time before starting treatment would actually mirror real life clinical settings. The impact of such waiting time on drop-out rates from treatment does, however not seem relevant here as such impact is relevant mostly for patients with severe comorbid conditions [94].

## Abbreviations

ANCOVA: analysis of covariance
BAI: Beck Anxiety Inventory
BDI: Beck Depression Inventory
BED: Binge eating disorder
BES: Binge Eating Scale
BMD: bone mineral density
BMI: body mass index
BN: Bulimia nervosa
CBT: cognitive behavioral therapy
CIA: clinical impairment assessment
CTX-1: carboxy-terminal collagen crosslinks
DSM-5: diagnostic and statistical manual of mental disorders, 5th edition
DT: dietary therapy
DXA: dual-energy x-ray absorptiometry
ED: eating disorders
EDE-q: Eating Disorder Questionnaire
EDI-3: Eating Disorder Inventory, version 3
EDS: Eating Disturbance Scale
FSH: follicle-stimulating hormone
GP: general practitioner
LH: luteinizing hormone
NSSS: Norwegian School of Sport Sciences
ORS: Outcome Rating Scale
OSTRC: Oslo Sport Trauma Research Center questionnaire on health problems
PE: physical exercise
PED-t: Physical exercise and dietary therapy
PTH: Parathyroid hormone
RM: repetition maximum
T3: triiodothyronine
T4: thyroxine
TFEQ-21: Three Factor Eating Questionnaire, 21 items version
TSH: thyroxine stimulating hormone
